# Supplementary material for: The Cysteine Protease Giardipain-1 from Giardia duodenalis Contributes to a Disruption of Intestinal Homeostasis
Source: Int J Mol Sci. 2022 Nov 7;23(21):13649. doi: 10.3390/ijms232113649 (PMC9655832; doi:10.3390/ijms232113649)
Supplement: Supplementary file 1 [file ijms-23-13649-s001.zip › ijms-1902726-supplementary.pdf]

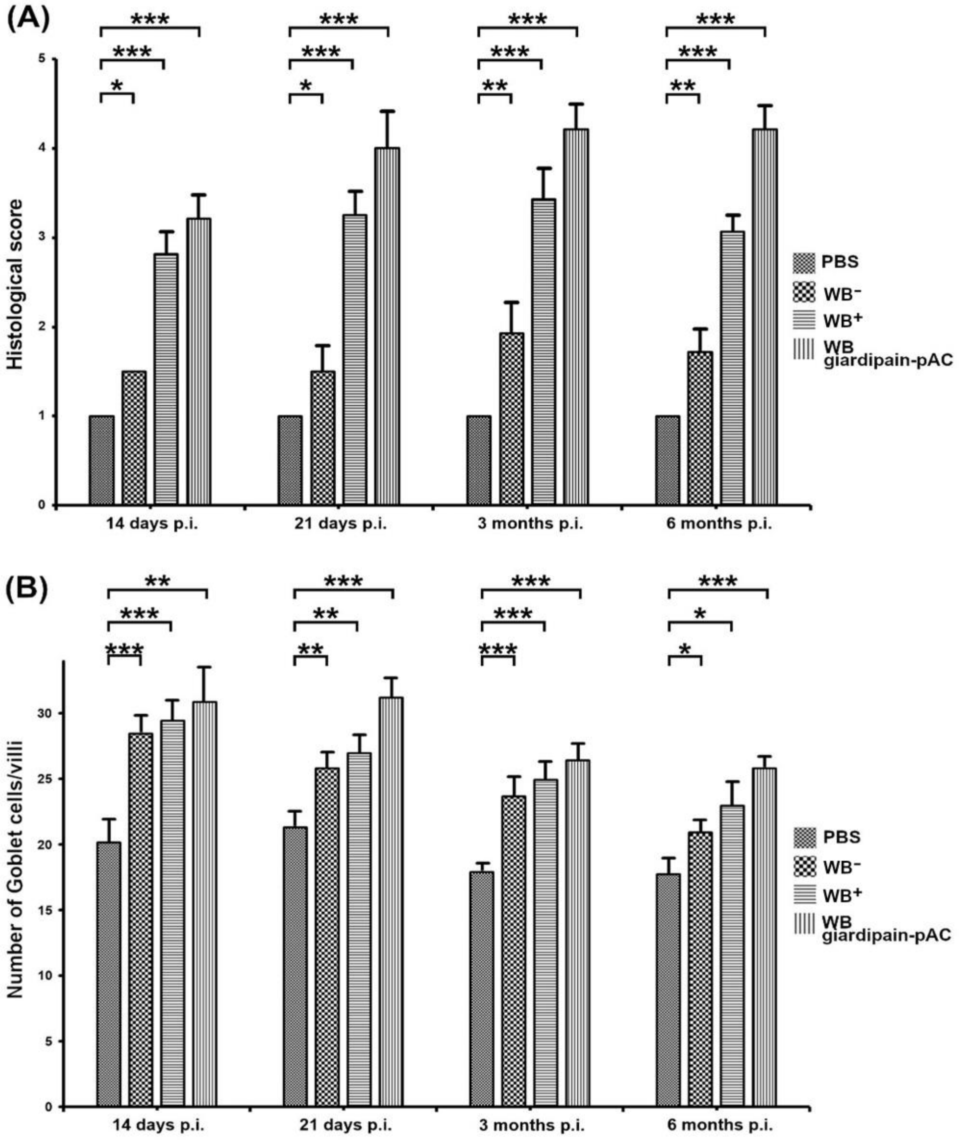

Supplementary Figure S1. A) Histological scores for jirds given PBS (control) or infected with WB+ or WB giardipain-pAC trophozoites for periods of 14 days, 21 days, 3 months and 6 months p.i. B) Quantitative analysis of the number of goblet cells in the villi of the duodenum sections of jirds that were given PBS (control) or infected with WB+ or WB giardipain-pAC. PAS-AB staining, 300 μm and 100 μm scale bar. Results are expressed as mean ± S.E. *p < 0.05, **p < 0.01, ***p < 0.001.
